# A method for selective and efficient isolation of gray matter astrocytes from the spinal cord of adult mice

**DOI:** 10.1186/s13041-024-01097-3

**Published:** 2024-05-21

**Authors:** Ryoma Iwasaki, Yuta Kohro, Makoto Tsuda

**Affiliations:** Department of Molecular and System Pharmacology, Graduate School of Pharmaceutical Sciences, Kyushu University, 3-1-1 Maidashi, Higashi-ku, Fukuoka, 812-8582 Japan

**Keywords:** Astrocytes, Spinal cord, ACSA-2 antibody, Cold-active protease, Gray matter, Fluorescence-activated cell sorting, DRAQ5

## Abstract

A growing body of evidence indicates intra- and inter-regional heterogeneity of astrocytes in the brain. However, because of a lack of an efficient method for isolating astrocytes from the spinal cord, little is known about how much spinal cord astrocytes are heterogeneous in adult mice. In this study, we developed a new method for isolating spinal astrocytes from adult mice using a cold-active protease from *Bacillus licheniformis* with an astrocyte cell surface antigen-2 (ACSA-2) antibody. Using fluorescence-activated cell sorting, isolated spinal ACSA-2^+^ cells were divided into two distinct populations, ACSA-2^high^ and ACSA-2^low^. By analyzing the expression of cell-type marker genes, the ACSA-2^high^ and ACSA-2^low^ populations were identified as astrocytes and ependymal cells, respectively. Furthermore, ACSA-2^high^ cells had mRNAs encoding genes that were abundantly expressed in the gray matter (GM) but not white matter astrocytes. By optimizing enzymatic isolation procedures, the yield of GM astrocytes also increased. Therefore, our newly established method enabled the selective and efficient isolation of GM astrocytes from the spinal cord of adult mice and may be useful for bulk- or single-cell RNA-sequencing under physiological and pathological conditions.

**Supplementary Information:**

The online version contains supplementary material available at 10.1186/s13041-024-01097-3.

## Introduction

Astrocytes, a type of glial cells, are distributed throughout the central nervous system and are known to play diverse roles in maintaining homeostasis [1]. For example, astrocytes modulate extracellular pH and ion and neurotransmitter concentrations through specific channels and transporters that affect neuronal functions in the brain and spinal cord [2]. Accumulating evidence indicates that astrocytes exhibit intra- and inter-regional heterogeneity in terms of molecular, morphological, and functional characteristics [3]. In particular, recent advanced technologies have shed light on astrocyte heterogeneity in individual brain regions (e.g., the hippocampus [4, 5], cortex [6, 7], hypothalamus [8], and amygdala [9]) and have categorized astrocytes into clusters based on their gene expression profiles. In the spinal cord, astrocytes are distributed in tile form in the white and gray matter (WM and GM, respectively), and their morphology [10] and gene or protein expression profiles [11] are quite different from those of brain astrocytes. Recently, we identified a population of spinal cord astrocytes defined by the expression of hairy and enhancer of split 5 (Hes5). Hes5^+^ astrocytes are selectively localized to the superficial laminae (laminae I–IIIo) of the spinal dorsal horn (SDH) and play a crucial role in modulating the processing of mechanical information from the periphery [12]. Furthermore, another astrocyte defined by the expression of GFP under the control of a lunatic fringe promoter was recently found in laminae III–IV [13]. Despite recent progress, there have been no reports on the comprehensive characterization of astrocytic heterogeneity in the spinal cord at the single-cell level, for example, using single-cell RNA-sequencing (scRNA-seq). One reason for this may be the lack of efficient methods for isolating spinal astrocytes from adult mice. Previously, spinal astrocytes have been isolated using papain-based tissue digestion. However, the yield of isolated astrocytes was much lower than that of brain astrocytes [14]. Furthermore, the conventional cell dissociation method using a protease requires heating, which has been reported to artificially induce or change the expression of genes, such as immediate early genes, in several brain cells including astrocytes [9].

To overcome these limitations, we used a cold-active protease from *Bacillus licheniformis* [15] with an astrocyte cell surface antigen-2 (ACSA-2) antibody [16] and efficiently isolated astrocytes from the mouse spinal cord in high yields using fluorescence-activated cell sorting (FACS). We also found that the cells in the ACSA-2^high^ fraction were GM astrocytes. Thus, our established method enables the selective and high-yield isolation of GM astrocytes from the spinal cord of adult mice and may be useful for further transcriptomic and epigenetic analyses at the bulk and single-cell levels to advance our understanding of astrocyte heterogeneity under physiological and pathological conditions.

## Results

### Selective immunolabeling of GM astrocytes in the spinal cord by ACSA-2 antibody

**Fig. 1:**
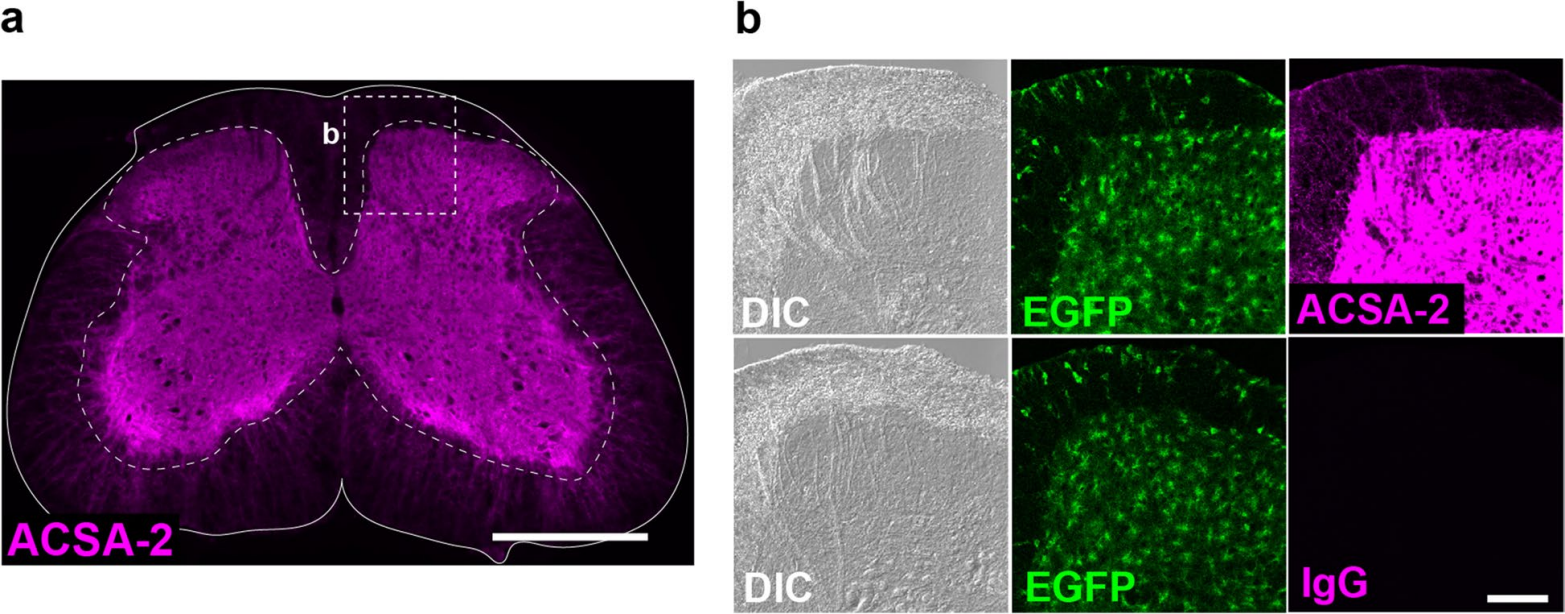
Specific ACSA-2-immunostaining in the gray matter (GM) of spinal cord in adult mice. **a** Immunolabeling with an ACSA-2 antibody in the fourth lumbar (L4) spinal cord of *Aldh1l1*-*EGFP* mice. The dashed line indicates the boundary between GM and white matter (WM). **b** Representative immunohistochemical images of ACSA-2 and matched IgG control antibodies in the spinal dorsal horn (SDH) of *Aldh1l1*-*EGFP* mice. The immunofluorescence intensities of ACSA-2 and IgG was enhanced to a saturated level in the GM by increasing the gain of image acquisition. Scale bars, 500 μm (**a**) and 100 μm (**b**)

*Aldh1l1-EGFP* mice, in which almost all astrocytes express EGFP reporter proteins [17], and an ACSA-2 antibody have been frequently used to isolate adult mouse brain astrocytes using FACS [4, 16, 18, 19]. To explore the best method of FACS isolation of spinal astrocytes in adult mice, we first compared the distribution patterns of EGFP^+^ and ACSA2^+^ cells using *Aldh1l1*-*EGFP* mice. EGFP^+^ cells in the lumbar spinal cord of *Aldh1l1*-*EGFP* mice were ubiquitously observed in both GM and WM (Additional file 1: Supplementary Fig. 1a). The fluorescence intensity of EGFP in individual cells was slightly higher in GM astrocytes than in WM astrocytes, and was not detected in wild-type (WT) mice (Additional file 1: Supplementary Fig. 1b). EGFP^+^ cells in the GM were co-labelled with the astrocytic marker SRY-related high-mobility group box 9 (SOX9) but not with SRY-related high-mobility group box 10 (SOX10, for oligodendrocyte lineage cells), neuronal nuclear antigen (NeuN, for neurons), or ionized calcium-binding adapter molecule 1 (IBA1, for microglia) (Additional file 1: Supplementary Fig. 1c). In the WM of the spinal cord, some EGFP^+^ cells were co-stained with SOX9 and others with SOX10 (Additional file 1: Supplementary Fig. 1d). In contrast, ACSA-2 immunofluorescence in the spinal cord was selectively observed in the GM (Fig. 1a). However, by increasing the gain of image acquisition (at a saturated level in the GM), weak immunofluorescence signals of ACSA-2 were detected in the WM, which was higher than that in the IgG control (Fig. 1b). These results suggest that the ACSA-2 antibody is a useful tool for selectively isolating GM astrocytes from the spinal cord of adult mice.

### Isolation of spinal astrocytes by FACS using ACSA-2 antibody

**Fig. 2:**
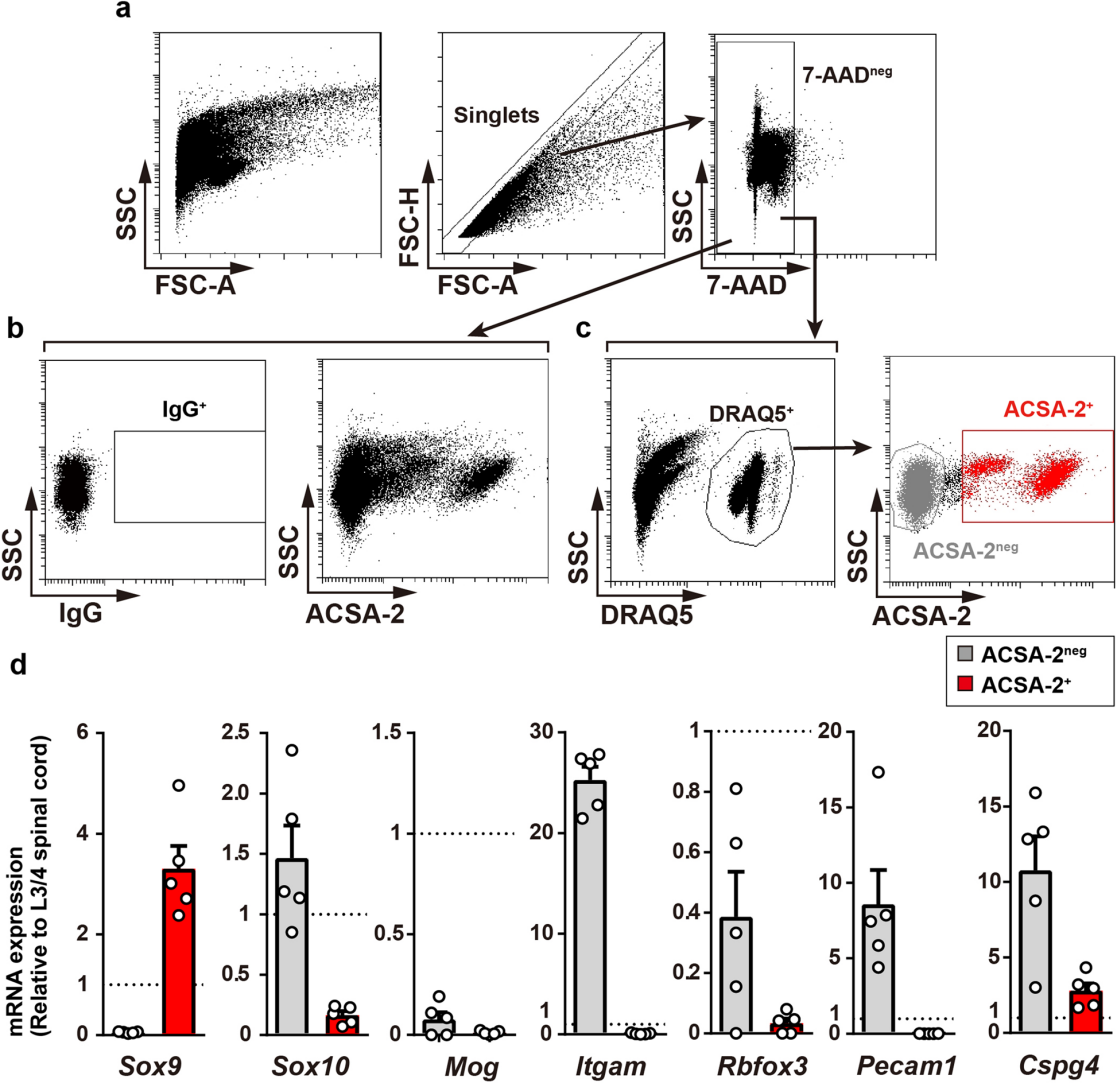
FACS isolation of ACSA-2^+^ cells from the lumbar spinal cord. **a**, **b**, **c** Gating strategy for collecting ACSA-2^+^ cells from total cells in the L3/4 spinal cord of adult mice stained with IgG-PE (**a**), ACSA2-PE (**b**), or ACSA-2-PE with DRAQ5 (**c**). ACSA-2^+^ cells were gated using singlets of 7-AAD^neg^ (live), DRAQ5^+^ (cell body), and ACSA-2^+^ . **d** Quantitative polymerase chain reaction (qPCR) analysis of cell-type specific markers in isolated ACSA-2^+^ and ACSA-2^neg^ cells (*n* = 5 mice). Values represent the relative ratio of mRNA (normalized to *Gapdh* mRNA) to the mean mRNA expression level of total cells in the L3/4 spinal cord sample. Data show mean ± SEM.

Papain is the most frequently used cysteine protease to dissociate brain and spinal cord tissues to isolate astrocytes [13, 18, 19]. Heating tissue samples is needed to achieve their highest enzymatic activity, but this has been shown to allow for artificial changes in gene expression in several cell types, including astrocytes [9]. Thus, we selected a cold-active serine protease purified from the psychrophilic soil bacterium *Bacillus licheniformis* [15]. The lumbar spinal cord tissues (the third and fourth lumbar (L3/4) segments) were treated with this enzyme at 4 °C for 20 min, myelin debris and oligodendrocytes were removed from the suspension using myelin removal beads (20 µL per one L3/4 spinal cord tissue), and ACSA-2^+^ cells were isolated by FACS (Fig. 2a-c). After doublet exclusion, 7-aminoactinomycin D (7-AAD)-negative viable cells immunolabeled with the ACSA-2 antibody, but not the IgG control, were selected as ACSA-2^+^ cells (Fig. 2b). As ACSA-2 recognizes the cell surface protein ATPase Na^+^/K^+^-transporting subunit beta 2 (ATP1B2), the fraction of ACSA-2^+^ cells may have included astrocytic process fragments. To purify the cell bodies of astrocytes, we further treated the ACSA-2 antibody-containing cell suspension with DRAQ5, a cell-permeable far-red fluorescent DNA dye [20] and found that ACSA-2^high^ and ACSA-2^low^ cells were clearly identifiable (Fig. 2c). Consistent with previous data [16], quantitative polymerase chain reaction (qPCR) analysis of the sample extracted from ACSA-2^+^ cells showed that astrocyte (*Sox9*), but not other cell-types markers (*Sox10* for oligodendrocyte lineage cells, *Mog* for mature oligodendrocytes, *Itgam* for microglia, *Rbfox3* for neurons and *Pecam1* for endothelial cells except *Cspg4* for perivascular cells and oligodendrocyte precursor cells) were enriched in ACSA-2^+^ population compared with the L3/4 spinal cord tissues (Fig. 2d). These results indicate that this protocol enables the isolation and purification of spinal astrocytes from adult mice.

### Highly efficient isolation of the GM astrocytes and ependymal cells from the mouse spinal cord

**Fig. 3:**
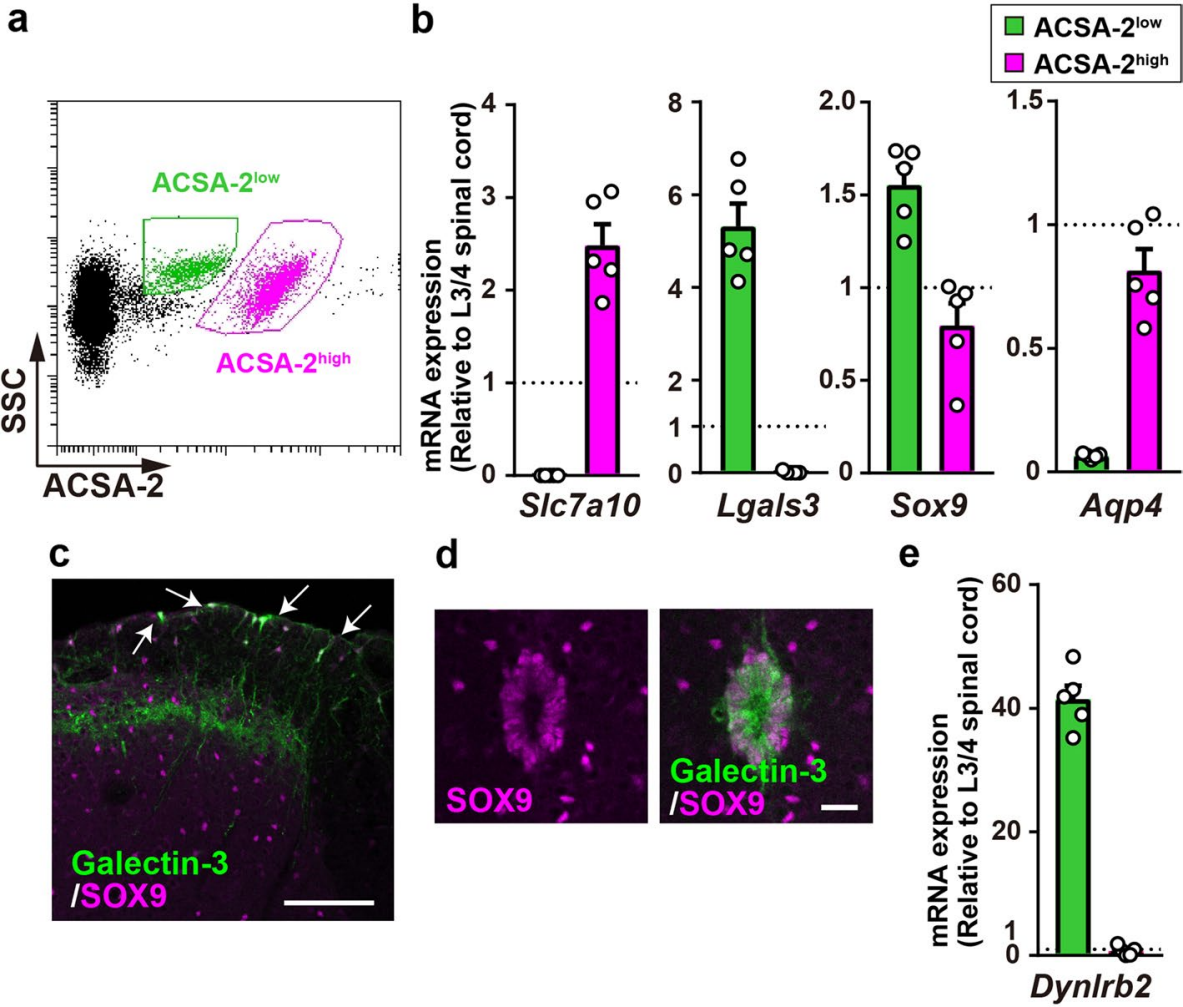
Identification of ACSA-2^high^ and ACSA-2^low^ cells as GM astrocytes and ependymal cells, respectively. **a** Representative scatter plots of ACSA-2^high^ and ACSA-2^low^ cells. **b** qPCR analysis of *Slc7a10*, *Lgals3*, *Sox9* and *Aqp4* mRNAs in fluorescence-activated cell sorting (FACS)-sorted ACSA-2^high^ and ACSA-2^low^ cells (*n *= 5 mice). **c**, **d** Immunostaining of galectin-3 (green) and SOX9 (magenta) in the SDH (**c**) and the central canal (**d**) of wild-type mice. **e** qPCR analysis of *Dynlrb2* mRNA in FACS-sorted ACSA-2^high^ and ACSA-2^low^ cells (*n* = 5 mice). Scale bars, 100 μm (**c**) and 20 μm (**d**). Data show mean ± SEM

Based on the differences in ACSA-2 immunofluorescence in the lumbar spinal cord (Fig. 1a, b), we speculated that the ACSA-2^high^ and ACSA-2^low^ populations (Fig. 3a) could be GM and WM spinal astrocytes, respectively. To characterize the populations of ACSA-2^high^ and ACSA-2^low^ cells, mRNA was extracted from these populations separately. The expression of alanine–serine–cysteine transporter-1 (ASC-1; encoded by *Slc7a10*), an Na^+^-independent amino acid transporter, is highly specific to GM astrocytes in the spinal cord [21, 22]. As expected, ACSA-2^high^ but not the ACSA-2^low^ population expressed *Slc7a10* (Fig. 3b). Galectin-3 (encoded by *Lgals3*) which is known to be expressed in WM astrocytes [23] was specifically detected in the ACSA-2^low^ population (Fig. 3b). However, in contrast to the expression of *Sox9*, *Aqp4* which is a marker of astrocytes, was much lower in ACSA-2^low^ cells than in ACSA-2^high^ cells (Fig. 3b). Immunohistochemical analysis showed that galectin-3 and SOX9 were expressed in WM astrocytes (Fig. 3c) and ependymal cells (Fig. 3d). Consistent with this result, ACSA-2^low^ but not ACSA-2^high^ cells highly expressed *Dynlrb2*, a marker of ependymal cells [24, 25] (Fig. 3e). By increasing the gain of image acquisition, weak immunofluorescence signals of ACSA-2 were also detected in the ependymal cells, the intensity of which was higher than that in IgG control (Additional file 1: Supplementary Fig. 2). These results suggest that the ACSA-2^high^ and ACSA-2^low^ populations are GM spinal astrocytes and ependymal cells, respectively.

### Optimized isolation procedure enhances the yield of GM spinal astrocytes

**Fig. 4:**
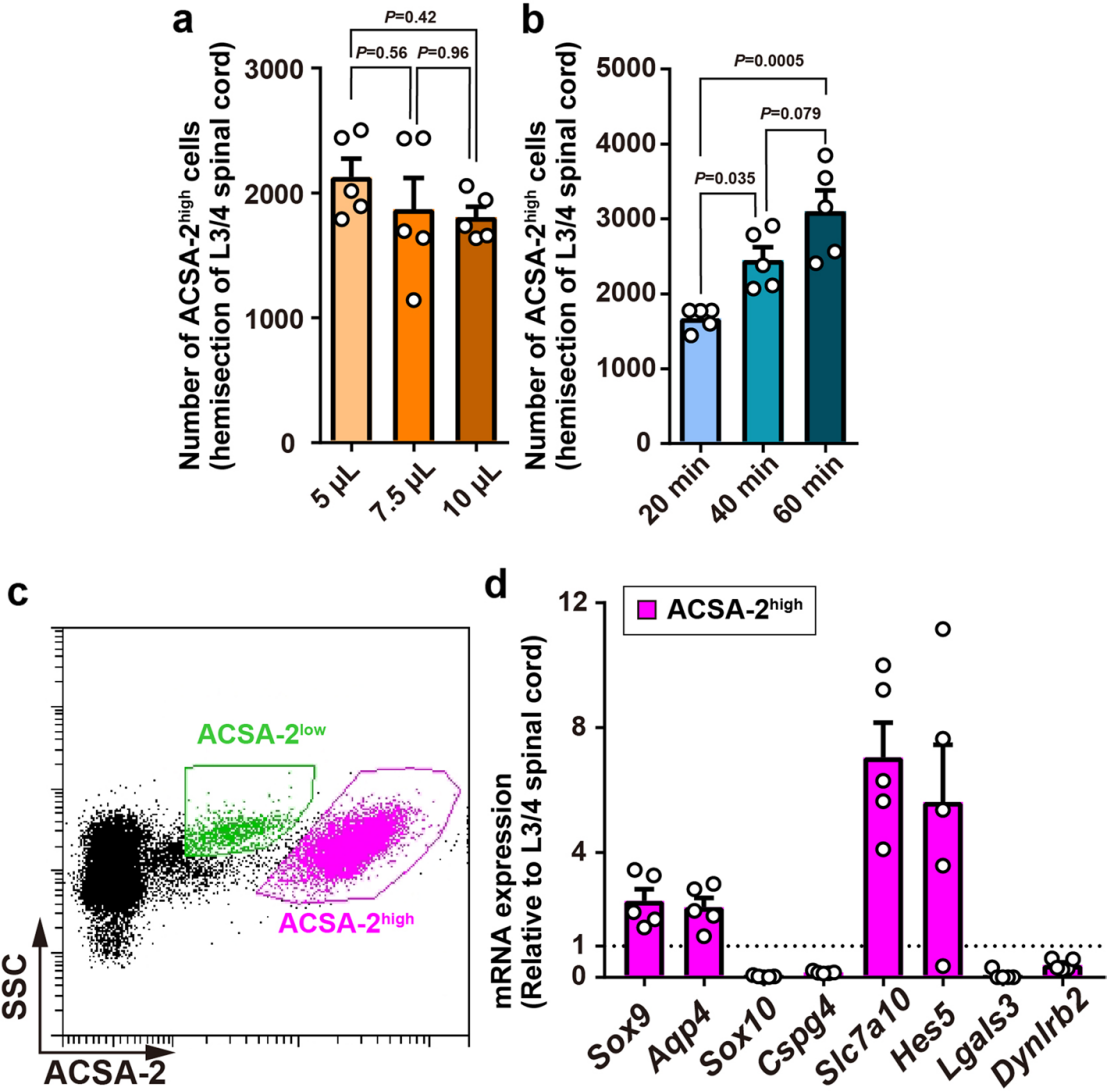
Optimization of tissue processing for enhancing the yield of spinal GM astrocytes.  **a**, **b** Effects of myelin removal (**a**) and enzymatic reaction (**b**) on the yield of isolated ACSA-2^high^ cells from the hemisection of the L3/4 spinal cord (*n* = 5 mice). **c** Representative FACS plots of cells stained with the ACSA-2 antibody under the optimized myelin removal and tissue dissection protocol. **d** qPCR analysis of cell type-specific markers in FACS-sorted ACSA-2^high^ cells (*n* = 5 mice). Data show mean ± SEM

To enhance the yield of ACSA-2^high^ population, we optimized the processes of myelin removal and enzymatic reactions. As the treatment with myelin removal beads has been reported to affect the yield of astrocytes [26], we tested different volumes of myelin removal beads (5, 7.5, or 10 µL per one hemi-section of the L3/4 spinal cord). A low volume of myelin removal beads slightly increased the number of ACSA-2^high^ cells (Fig. 4a). Furthermore, as a longer duration of enzymatic treatment allowed for effective dissociation at a lower temperature [9], we compared the effect of three different dissociation periods (20, 40, or 60 min) and found that enzymatic dissociation for 60 min enabled the isolation of the highest number of ACSA-2^high^ cells (Fig. 4b). In contrast to the ACSA-2^high^ cells, these procedures had non-significant effects on the yield of ACSA-2^low^ cells (Additional file 1: Supplementary Fig. 3a, b). Under this procedure (myelin removal beads 5 µL, and enzymatic dissociation for 60 min), we confirmed that FACS-purified ACSA-2^high^ cells (7079 ± 269 cells per L3/4 spinal cord tissue, *n* = 5) highly expressed astrocytic markers (*Sox9* and *Aqp4*) and exhibited low expression of *Sox10* and *Cspg4* (Fig. 4c, d). Furthermore, the mRNAs of *Slc7a10* and *Hes5* (a marker for our identified subpopulation of astrocytes in the SDH) were enriched in the ACSA-2^high^ population (Fig. 4d). In contrast, *Sox9*, *Lgals3*, and *Dynlrb2* were enriched in the ACSA-2^low^ population (1355 ± 56 cells per the L3/4 spinal cord, *n* = 5) (Additional file 1: Supplementary Fig. 3c). These results indicate that our established method enables the highly effective and selective isolation of GM astrocytes (and ependymal cells) from the spinal cord of adult mice.

## Discussion

Increasing evidence indicates molecular and functional heterogeneity of astrocytes in the brain under physiological and pathological conditions [3]. However, the extent to which spinal cord astrocytes are heterogeneous remains to be fully understood because there is a lack of efficient methods to sort spinal cord astrocytes, which hinders a comprehensive analysis of astrocytic heterogeneity, such as scRNA-seq. Here, we established an optimal method for the efficient and high-yield isolation of GM astrocytes from the spinal cord of adult mice. The key to this achievement was to employ cold-active protease-based cell dissociation, cell body isolation using the DNA dye DRAQ5, and cell labeling using an ACSA-2 antibody. Using DRAQ5, the indistinct ACSA-2^+^ cell population was divided into two distinguished populations: ACSA-2^high^ and ACSA-2^low^. Thus, the ACSA-2^+^ fraction may include process fragments of astrocytes that were produced by the tissue dissociation procedure. Furthermore, by analyzing gene expression, we demonstrated for the first time that ACSA-2^high^ cells in the spinal cord of adult mice are GM astrocytes, enabling GM astrocyte-selective analyses. Moreover, our method achieved a high-yield isolation of GM astrocytes by optimizing the protocols for enzymatic tissue dissociation and myelin removal from cell suspensions. The optimized protocol yielded approximately 7,000 cells per L3/4 spinal cord from one mouse, which is much higher than the yield in a previous report using the cysteine protease papain (approximately 9,000 cells per whole spinal cord tissue from one mouse). The reason for this difference remains unclear. However, the serine protease used in our study may be suitable for cell dissociation to form a single-cell suspension from the spinal cord tissues of adult mice.

The process in which oligodendrocytes are removed from cell suspension by myelin removal beads is also important for efficient astrocyte purification using ACSA-2 antibody because, in addition to astrocytes, *Atp1b2* mRNA has been reported to be expressed in *Cspg4*^+^ or O1^+^ cells, which are considered oligodendrocyte precursors and mature oligodendrocytes, respectively [4, 16]. Compared to *Sox10* mRNA whose expression level was low in the ACSA-2^+^ population (Fig. 2d), these cells expressed *Cspg4* mRNA, a marker of oligodendrocyte precursor cells and perivascular cells. However, considering our further data showing that ACSA-2^high^ and ACSA-2^low^ cells had extremely low or low levels, respectively, of *Cspg4* mRNA (Fig. 4d), *Cspg4* expression detected in the ACSA-2^+^ population could be due to the inclusion of a few pericytes. In fact, scRNA-seq has revealed that brain cells isolated using the ACSA-2 antibody include mural cells [4] and astrocytes and pericytes in the spinal cord express *Atp1b2* [27].

An unexpected finding in this study was that cells in the ACSA-2^low^ fraction were ependymal cells, but not astrocytes (Fig. 3 and Additional file 1: Supplementary Figs. 2, 3). In fact, ACSA-2^low^ cells did not express the astrocytic gene *Aqp4* but did *Dynlrb2* which is a marker of ependymal cells. Other astrocytic genes, *Sox9* and *Lgals3* were detected in ACSA-2^low^ cells, but SOX9 and galectin-3 were also found in ependymal cells of the central canal, which is consistent with previous data. Considering our data, ACSA-2^+^ cells isolated using only ACSA-2 magnetic beads could be a mixture of astrocytes and ependymal cells; thus, caution should be exercised when interpreting data obtained from non-fractionated ACSA-2^+^ cells. However, because, to our knowledge, there is currently no efficient isolation method for ependymal cells by antibody-based FACS in non-transgenic WT mice, our method could also be useful for ependymal cell isolation, although there is one previous report that isolated ependymal cells and investigated their heterogeneity using a reporter protein-expressing transgenic mouse line [25].

A limitation of this study was that WM astrocytes were not isolated from the spinal cord using our method. A similar difficulty was also reported in the optic nerve, which may be related to the dense myelination of these tissues [28]. Fluorescence-activated nuclei sorting [29] or Ribo Tag immunoprecipitation [30] may be used to overcome this limitation. Another limitation was that this study examined only adult mice because the expression of ATP1B2 in the brain is detectable from embryonic day 18.5 (E18.5) [16]; however, its expression level and cell-type specificity in the spinal cord remain to be determined. Further investigations are needed to clarify whether the current protocol applies to pre- and postnatal or aged mice.

An advantage of our method is to apply for isolating spinal astrocytes from genetically modified mice (e.g., cell-type-specific conditional knockout mice or genetic animal models of disease) because there is no need to cross additional transgenic mice, such as *Aldh1l1-EGFP* mice. Recent single-cell RNA-seq analysis revealed that *Atp1b2* is predominantly expressed in astrocytes of the marmoset [31], developing [32] and adult human spinal cords [33]. Therefore, our established method can be adapted to other species. Among more important challenges are to determine whether there is heterogeneity within the ACSA-2^high^ population in the spinal cord by scRNA-seq analysis of isolated spinal astrocytes using our established method, since a recent scRNA-seq analysis of ACSA-2^+^ brain astrocytes has revealed that these cells are divided into several populations [4]. Furthermore, previous papers have shown that cultured astrocytes acutely isolated from mice and human have abilities to secrete molecules, promote neuron survival and respond to various stimuli [34–36]. Thus, the functional analysis of isolated spinal astrocytes using our established method is also an important subject. Thus, our technique will be useful for transcriptomic and epigenetic analyses at bulk and single-cell levels, deepening our understanding of spinal astrocyte heterogeneity under physiological and pathological conditions.

## Methods

### Animals

Male C57BL/6 mice (CLEA Japan, Tokyo, Japan) were used. Male and female *Aldh1l1-EGFP* mice (B6;FVB-Tg(Aldh1l1-EGFP/Rpl10a)JD130Htz/J) [17] were purchased from The Jackson laboratory. All mice used were 8–10 weeks of age at the start of each experiment and were housed at 22 ± 1 °C with a 12-h light-dark cycle. All animals were fed food and water *ad libitum*. All animals were housed in standard polycarbonate cages in groups of same-sex littermates. All animal experiments were conducted according to relevant national and international guidelines contained in the ‘Act on Welfare and Management of Animals’ (Ministry of Environment of Japan) and ‘Regulation of Laboratory Animals’ (Kyushu University) and under the protocols approved by the Institutional Animal Care and Use committee review panels at Kyushu University.

### Immunohistochemistry

Mice were deeply anesthetized with intraperitoneal (i.p.) injection of pentobarbital and transcardially perfused with phosphate-buffered saline (PBS) followed by ice-cold 4% paraformaldehyde (PFA)/PBS. The transverse fourth lumbar (L4) segments of spinal cord was removed, postfixed in the same fixative for 3 h at 4°C. According to a method of our previous paper [12], transverse sections of L4 spinal cord (30 µm) were incubated for 48 h at 4°C with primary antibodies for ACSA-2-PE (1:50, 130-116-244, Miltenyi Biotec, Bergisch-Gladbach, Germany), IgG-PE (1:50, 130-113-450, Miltenyi Biotec, Bergisch-Gladbach, Germany), polyclonal goat anti-SOX9 (1:2000, R&D Systems, MN, USA), polyclonal goat anti-SOX10 (1:500, AF2864, R&D Systems, MN, USA), monoclonal rabbit anti-NeuN (1:2000, ab17748, abcam, Cambridg, UK), polyclonal guinea pig anti-IBA1 (1:2000, 234 00, Synaptic Systems, Goettingen, Germany) and monoclonal rat anti-galectin-3 (1:500, CL8942AP, Cedarlane Labs, Burlington, Canada). Tissue sections were incubated with secondary antibodies conjugated to Alexa Fluor 488 (1:1000, A-21208, Thermo Fisher), Alexa Fluor 546 (1:1000, A-11056, Thermo Fisher) or DyLight 405 (1:1000, 711-475-152 or 706-475-148, Jackson ImmunoResearch) and mounted with or without 4’,6-diamidino-2-phenylindole (DAPI; Vector Laboratories, USA). Three sections from one tissue were randomly selected and images were taken using an LSM700 Imaging System (Zen 2012, Carl Zeiss) and BZ-X800 fluorescence microscope (Keyence Corporation, Osaka, Japan).

### Fluorescence activated cell sorting (FACS)

Mice were deeply anesthetized by i.p. injection of pentobarbital and perfused transcardially with ice-cold PBS. The L3/4 spinal cord was rapidly removed from the vertebral column and placed into ice-cold Hanks’ balanced salt solution (HBSS) without Ca^2+^, Mg^2+^ (HBSS (-)). The dura and pia matter and dorsal/ventral roots were carefully removed from the spinal cord, and spinal cord tissue was cut into 8 pieces. The spinal tissue pieces were treated with ice-cold 1 mL enzymatic solution [10 mg/mL of protease from *Bacillus licheniformis* enzyme (Sigma-Aldrich, P5380) with 125 units/mL of DNaseI (Worthington Biochemical Corporation) and 5 mM CaCl_2_] in HBSS (-) for 10 or 30 min at 4 °C. The tissues were homogenized by passing through a 23G needle attached with 1 mL syringe and were further incubated for 10 or 30 min at 4 °C. After that, the tissues were homogenized by passing through a 26G needle, and the enzymatic reaction was stopped by HBSS with Ca^2+^, Mg^2+^. After centrifugation (1000 × g, 5 min, 4 °C), the cells were resuspended in HBSS (-) with 0.5% BSA, 2mM EDTA and 10 or 20 µL Myelin Removal Beads II (Milteny Biotec) for 15 min at 4 °C to remove myelin debris and oligodendrocytes from the suspension. In the case of the hemi-section of the L3/4 spinal cord, the tissue pieces were treated with ice-cold 0.5 mL enzymatic solution for 20, 40 or 60 min at 4 °C and the cell suspension was incubated with 5, 7.5 or 10 µL Myelin Removal Beads II. After centrifugation (300 × g, 10 min, 4 °C), the cells were resuspended in HBSS (-) with 0.5% BSA and 2mM EDTA, and passed through a magnetic cell sorting (MACS) LS column (Milteny Biotec) according to the manufacturer’s protocol at 4 °C. After centrifugation (300 × g, 10 min, 4 °C), the cell suspension was blocked by incubating with Fc Block (1:200, 553142, BD Biosciences) for 5 min and immunostained with IgG-PE (1:200, 130-113-450, Miltenyi Biotec) or ACSA2-PE (1:200, 130-116-244, Miltenyi Biotec) for 10 min at 4 °C in the dark. After washing, the cell suspension was treated with 7-aminoactinomycin D (7-AAD; 1:40, 00-6993-50, eBioscience) and DRAQ5 (1:666, 130-117-343, Milteny Biotec) and incubated for at least 15 min on ice for viability and targeting cells with nucleus staining prior to cell sorting. 7-AAD^neg^ DRAQ5^+^ ACSA-2^+^ cells were gated as live cells and sorted depending on ACSA-2 fluorescent intensity using cytoFLEX SRT (Beckman coulter, CA, USA).

### Quantitative polymerase chain reaction (qPCR)

The sorted cells were subjected total RNA extraction using the Quick-RNA Micro-Prep kit according to the manufacturer’s protocol (Zymo Research, CA, USA). The extracted RNA from the sorted cells was transferred to reverse transcriptional reaction with Prime Script reverse transcriptase (Takara, Japan). qPCR was performed with Premix Ex Taq (Takara, Japan) using QuantStudio3 (Applied Biosystems, MA, USA). Expression levels were normalized to the value for *Gapdh* and values below detection level were regarded as zero. The following primers and probes were obtained from Integrated DNA Technologies (IA, USA): *Sox9* (NM_011448; SRY (sex determining region Y)-box 9), *Sox10* (NM_011437; SRY (sex determining region Y)-box 10), *Mog* (NM_010814; myelin oligodendrocyte glycoprotein), *Itgam* (NM_001082960; integrin subunit alpha M), *Rbfox3* (NM_001039168; RNA binding protein, fox-1 homolog (C. elegans) 3), *Pecam1* (NM_001032378; platelet/endothelial cell adhesion molecule 1), *Cspg4* (NM_139001; chondroitin sulfate proteoglycan 4), *Aqp4* (NM_009700; aquaporin 4), *Slc7a10* (NM_017394; solute carrier family 7 (cationic amino acid transporter, y+ system), member 10), *Hes5* (NM_010419; hairy and enhancer of split 5) and *Dynlrb2* (NM_029297; dynein light chain roadblock-type 2). The sequences of TaqMan primer pairs and probe were described below.

*Gapdh*: 5′-FAM-ACCACCAACTGCTTAGCCCCCCTG-TAMRA-3′ (probe), 5′-TGCCCCCATGTTTGTGATG-3′ (forward primer), 5′-GGCATGGACTGTGGTCATGA-3′ (reverse primer).

*Lgals3*: 5’-FAM-CCCGCTGGACCACTGACGGTG-TAMRA-3’ (probe), 5’-TCCTGCTGCTGGCCCTTAT-3’ (forward primer), 5’-TTCACTGTGCCCATGATTGTG-3’ (reverse primer).

### Statistical analysis

Quantitative data were expressed as the means ± SEM. Data were analyzed by one-way analysis of variance (ANOVA) with the post hoc Tukey’s multiple comparisons test (Fig. 4a, b and Additional file 1: Supplementary Fig. 3a), Kruskal–Wallis test with the post hoc Dunn’s multiple comparisons test (Additional file 1: Supplementary Fig. 3b), as appropriate, after determining the normality (Shapiro–Wilk test) and variance (F-test or Brown–Forsythe test) of the experimental data. Statistical analyses were performed using Prism 7 (GraphPad, CA, USA). Values were considered significantly different at *P* < 0.05.

### Supplementary Information

Additional file 1: Supplementary Fig. 1. EGFP expression in the GM and WM of the spinal cord in adult *Aldh1l1-EGFP* mice. a, EGFP expression in the L4 spinal cord of *Aldh1l1-EGFP* mice. b, Representative immunohistochemical images of EGFP and SOX9 (magenta) in the SDH of *Aldh1l1-EGFP* and wild-type mice. c, d, Immunohistochemical identification of EGFP-expressing cells using cell-type markers (magenta; c SOX9, SOX10, NeuN, or IBA1; d SOX9 and SOX10) in the GM (c) and WM (d) of L4-SDH sections. Scale bars, 500 μm (a), 100 μm (b) or 20 μm (c, d). Supplementary Fig. 2. ACSA-2-immunostaining in the central canal (CC) of spinal cord in adult mice. Representative immunohistochemical images of ACSA-2 and matched IgG control antibodies with SOX9 (magenta, ependymal cells) at the CC of wild-type mice. The immunofluorescence intensities of ACSA-2 and IgG was enhanced to a saturated level in the GM by increasing the gain of image acquisition. Dashed lines indicate the boundary between the CC and GM. Scale bar, 20 μm. Supplementary Fig. 3. Effect of optimized tissue processing on the yield of isolating ependymal cells from spinal cord. a, b, Effects of myelin removal (a) and enzymatic reaction (b) on the yield of isolated ACSA-2^low^ cells from the hemisection of the L3/4 spinal cord (*n *= 5 mice). c, qPCR analysis of cell type-specific markers in FACS-sorted ACSA-2^low^ cells (*n* = 5 mice). Data show mean ± SEM.

## Data Availability

Requests for materials and correspondence should be addressed to M.T. (tsuda@phar.kyushu-u.ac.jp).

## Abbreviations

7-AAD: 7-Aminoactinomycin D
ACSA-2: Astrocyte cell surface antigen-2
ASC1: Alanine–serine–cysteine transporter-1
ATP1B2: ATPase Na^+^/K^+^ transporting subunit beta 2
FACS: Fluorescence-activated cell sorting
GM: Gray matter
HBSS: Hanks’ balanced salt solution
Hes5: Hairy and enhancer of split 5
IBA1: Ionized calcium-binding adapter molecule 1
MACS: Magnetic cell sorting
NeuN: Neuronal nuclear antigen
PBS: Phosphate-buffered saline
PFA: Paraformaldehyde
qPCR: Quantitative polymerase chain reaction
scRNA-seq: Single-cell RNA-sequencing
SDH: Spinal dorsal horn
SOX9: SRY-related high-mobility group box 9
SOX10: SRY-related high-mobility group box 10
WM: White matter
WT: Wild-type
