# Supplementary figures and images for: A method for selective and efficient isolation of gray matter astrocytes from the spinal cord of adult mice
Source: Mol Brain. 2024 May 21;17:25. doi: 10.1186/s13041-024-01097-3 (PMC11106874; doi:10.1186/s13041-024-01097-3)

**a**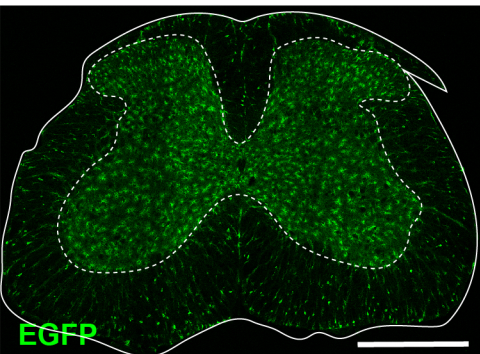**b***Aldh111-EGFP*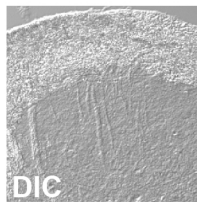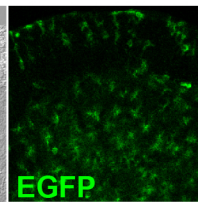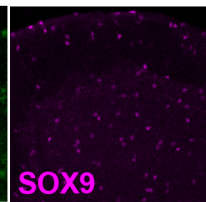

WT

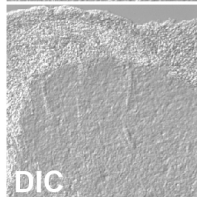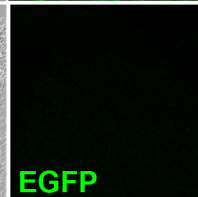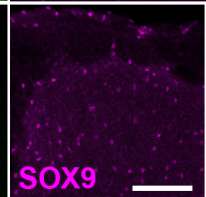**c**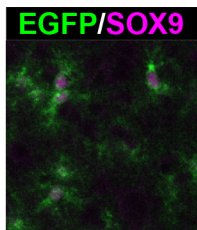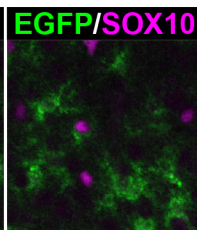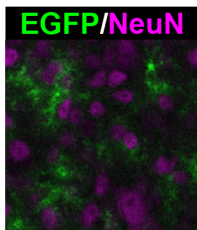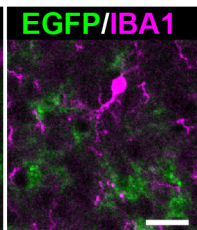**d**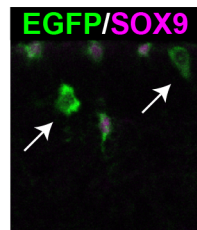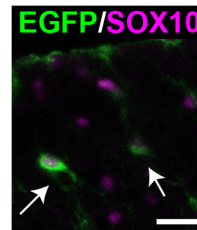

**Supplementary Fig.1**

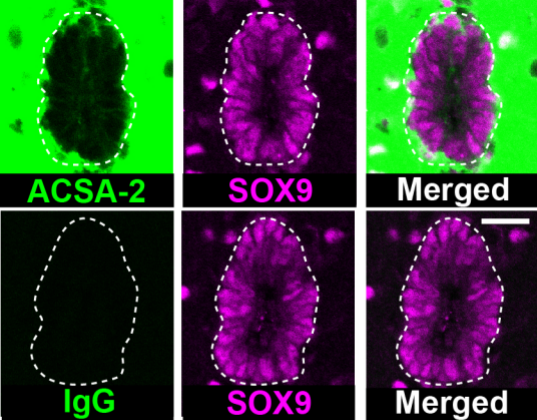

**Supplementary Fig. 2**

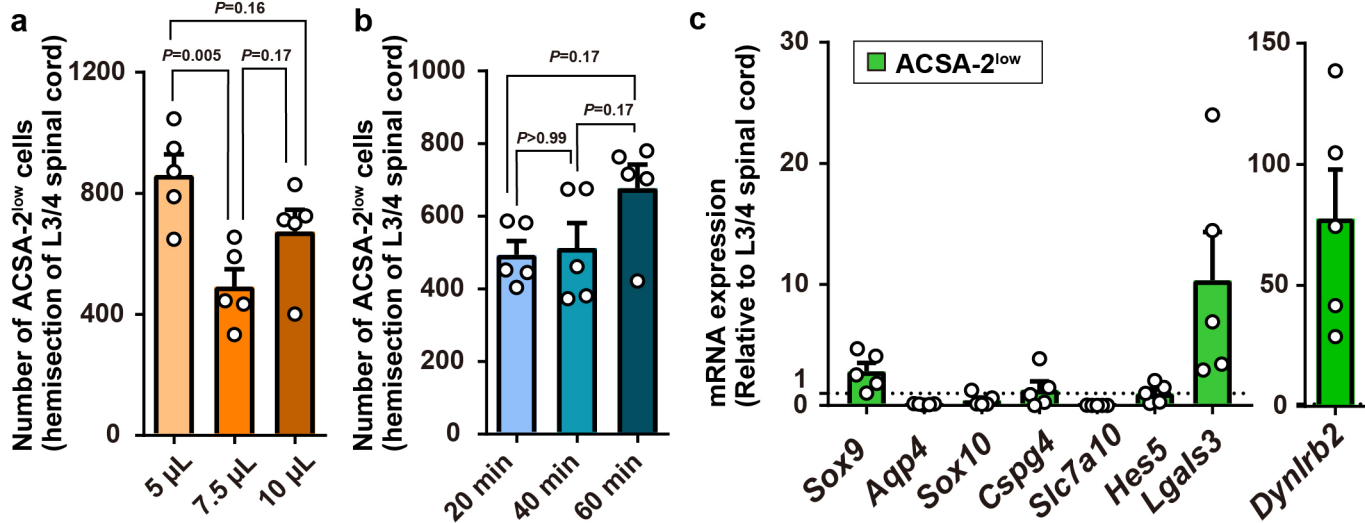

**Supplementary Fig. 3**

Supplement: Supplementary file 1 — Additional file 1: Supplementary Fig. 1. EGFP expression in the GM and WM of the spinal cord in adult Aldh1l1-EGFP mice. a, EGFP expression in the L4 spinal cord of Aldh1l1-EGFP mice. b, Representative immunohistochemical images of EGFP and SOX9 (magenta) in the SDH of Aldh1l1-EGFP and wild-type mice. c, d, Immunohistochemical identification of EGFP-expressing cells using cell-type markers (magenta; c SOX9, SOX10, NeuN, or IBA1; d SOX9 and SOX10) in the GM (c) and WM (d) of L4-SDH sections. Scale bars, 500 μm (a), 100 μm (b) or 20 μm (c, d). Supplementary Fig. 2. ACSA-2-immunostaining in the central canal (CC) of spinal cord in adult mice. Representative immunohistochemical images of ACSA-2 and matched IgG control antibodies with SOX9 (magenta, ependymal cells) at the CC of wild-type mice. The immunofluorescence intensities of ACSA-2 and IgG was enhanced to a saturated level in the GM by increasing the gain of image acquisition. Dashed lines indicate the boundary between the CC and GM. Scale bar, 20 μm. Supplementary Fig. 3. Effect of optimized tissue processing on the yield of isolating ependymal cells from spinal cord. a, b, Effects of myelin removal (a) and enzymatic reaction (b) on the yield of isolated ACSA-2low cells from the hemisection of the L3/4 spinal cord (n = 5 mice). c, qPCR analysis of cell type-specific markers in FACS-sorted ACSA-2low cells (n = 5 mice). Data show mean ± SEM. [file 13041_2024_1097_MOESM1_ESM.pdf]
